# Cryopreservation of rat embryos at all developmental stages by small-volume vitrification procedure and rapid warming in cryotubes

**DOI:** 10.1038/s41598-023-47394-0

**Published:** 2023-11-27

**Authors:** Shinsuke Seki, Toshiaki Kawabe, Wataru Yamazaki, Kazuaki Matsumura, Takanori Oikawa, Takahiro Obata, Misako Higashiya, Megumi Yano, Tomoo Eto

**Affiliations:** 1 Experimental Animal Division, Bioscience Education and Research Support Center, Akita University, 1-1-1 Hondo, Akita, Akita 010-8543 Japan; 2 ARK Resource Co., Ltd., 456 Osozu, Misato-machi, Shimomashiki-gun, Kumamoto, 861-4401 Japan; 3 School of Materials Science, Japan Advanced Institute of Science and Technology, 1-1 Asahi-dai, Nomi, Ishikawa 923-1292 Japan; 4 Central Institute for Experimental Animals, 3-25-12 Tonomachi, Kawasaki-ku, Kawasaki, 210-0821 Japan

**Keywords:** Cell biology, Developmental biology

## Abstract

Intracellular ice formation during cryopreservation is lethal to the cell, including during warming. Here, we examined the effect of sample volume and warming rate on the cryopreservation success of 1-cell rat embryos based on successful development into blastocysts in vitro and to term in vivo following embryo transfer. Embryos were equilibrated in 5% propylene glycol solution for 10 min, held for 40 s at 0 °C in cryopreservation solution (5%PG + PEPeS), and cooled by immersion in liquid nitrogen. When 1-cell embryos were cryopreserved in a volume of 30–100 μL at a cooling rate of 5830–7160 °C/min and warmed at 35,480–49,400 °C/min by adding 1 mL of 0.3 M sucrose solution at 50 °C, 17.3–28.8% developed into blastocysts, compared with 57.0% of untreated embryos. However, when 1-cell embryos were cryopreserved in a smaller volume of 15 μl at 7950 °C/min and warmed at 68,850 °C/min, 58.8 ± 10.6% developed into blastocysts and 50.0 ± 7.4% developed to term, comparable to that of non-treated embryos (57.0 ± 5.4% and 51.4 ± 3.1%, respectively). Cryopreserved embryos at other developmental stages also showed high in vitro culture potential similar to that of the control. Using a conventional cryotube and a small-volume vitrification procedure with rapid warming, we achieved high levels of subsequent rat embryonic development at all developmental stages.

## Introduction

The cryopreservation of gametes and embryos is an indispensable and important technology in human assisted reproductive technology (ART) and the preservation of genetic material of laboratory animals in gene banks. Cryopreservation is a cost-effective means for maintaining laboratory mammalian embryos, including genetically modified and transgenic lines of various mammals, such as mice and rats, for research purposes. Within the process of cryopreserving oocytes and embryos, the formation of even a small amount of ice within the cell is lethal. To counteract intracellular ice formation (IIF), two strategies were developed: slow freezing^1^ and vitrification^2^. Vitrification is widely used owing to its simplicity and high survivability. When cells are vitrified with minimal supercooling, close to their equilibrium state, ice formation remains entirely absent during both cooling and warming. However, in most instances, supercooling is not minimized, resulting in the formation of invisible ice crystals within the cells during cooling. These minuscule ice crystals pose no immediate injury if they remain minute, but during the warming process, they have the potential to recrystallize into larger, lethal intracellular ice crystals due to differences in surface free energy, ultimately leading to cellular damage^3^.

Research in the field of vitrification has primarily focused on the critical role of the cooling rate in preventing IIF with a few exceptions^4,5^. To prevent IIF during cooling, ultra-rapid vitrification has been introduced, utilizing small-scale tools, such as electron microscope grids^6^, open-pulled straws^7^, cryoloops^8^, and Cryotops^9^. These specialized tools enable the manipulation of oocytes and embryos within a very small volume (~ 0.1 μL) of vitrification solution, resulting in the attainment of extremely high cooling rates (> 10,000 °C/min). The first report describing the use of ultra-rapid vitrification was that of bovine oocyte vitrification with the aim of circumventing chilling injury around 0 °C. Ultra-rapid vitrification has also been applied for the cryopreservation of mammalian oocytes and embryos with the aim of preventing IIF during cooling.

In vitrification, it is believed that cells must be cooled rapidly and the vitrification solution in which cells are suspended must include a high concentration of cell-permeating cryoprotectants to counteract IIF. However, our research findings regarding the vitrification of mouse oocytes/embryos are not consistent with those beliefs. We have found that rapid warming has a more dominant effect on the survival of vitrified mouse oocytes and 8-cell mouse embryos than either rapid cooling or a high concentration of cryoprotectants^10–12^. Furthermore, it has been demonstrated that mouse oocytes and embryos can be cryopreserved without the necessity for cryoprotectant permeation, thanks to ultra-rapid warming achieved using laser pulses^13^. Therefore, we hypothesized that the high survival rate of vitrified mammalian oocytes/embryos is derived more from rapid warming than from rapid cooling, and a high concentration of cryoprotectants.

Laboratory rats (*Rattus norvegicus*) are characterized by their large size and ease of handling, and they are commonly used in animal experiments to study behavior, psychology, nutrition, endocrinology, and other research areas. Moreover, comprehensive database platforms related to laboratory rats have been developed, such as the Rat Genome Database in the USA^14^ and the National BioResource Project-Rat in Japan^15,16^. Rats are important models in various fields of biomedical and biological research, contributing to the understanding of the mechanisms of human disease and physiology^17^. In recent years, genome editing, using the CRISPR/Cas9 system, and microinjection or electroporation, has been used to develop knockout/knock-in animals, including animals other than mice, such as rats^18–20^. Until recently, reproductive engineering technology was not as fully developed in rats as it was in mice. However, the production of knockout/knock-in rats using in vitro fertilized embryos^21^, effective sperm cryopreservation methods and in vitro fertilization methods have been reported^22^. Because rats are 10-times larger than mice, it is not easy to simultaneously prepare animals for embryo collection, recipient mothers for embryo transfer, and foster mothers to raise pups delivered by caesarean section. Such difficulties could be reduced if cryopreserved 1-cell embryos could be thawed and supplied for genome editing experiments at any time. The demonstration of cryopreservation of 1-cell rat embryos with high survival rates is limited to a single study using a small Cryotop device for ultra-rapid vitrification^23^. These devices are expensive, and only 5–10 embryos can be cryopreserved using a single Cryotop. When genome editing experiments are performed, 12–16 genome-edited embryos are transferred to one rat and 5–10 fetuses are obtained. Therefore, it is desirable to cryopreserve 20–100 embryos using one device, such as a cryotube. Because rapid warming is important for vitrification which we have demonstrated, we hypothesized that rat embryos can be cryopreserved in a cryotube if warming is rapid. Therefore, using conventional cryotubes, commercially available vitrification solutions and laboratory pipettes that anyone can use, we examined whether it is possible to develop a cryopreservation method for 1-cell rat embryos only by rapid warming.

## Results

### Effect of warming rate on survival of 1-cell rat embryos

**Figure 1:**
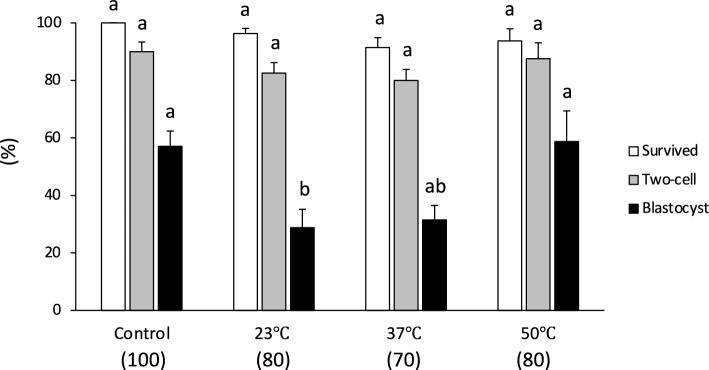
Survival and in vitro development of vitrified one-cell embryos (15 μL sample volume) warmed with sucrose solution at 23, 37, or 50 °C. The warming rates were 29,930 °C/min, 38,780 °C/min, and 68,850 °C/min, respectively. Each bar indicates the percentage of embryos surviving (open bars), developing to the two-cell stage (shaded bars), and developing to the blastocyst stage (closed bars) after cryopreservation (N = 8–10 experimental runs); numbers in parentheses indicate the number of one-cell embryos examined. Data are shown as means ± SEM. Different lowercase letters indicate a significant difference at *P* < 0.05.

To determine the effect of warming following cryopreservation of rat 1-cell embryos, cryotubes containing 15 μL samples were cooled at a rate of 7950 °C/min and then warmed at a rate of 27,300, 36,700, or 68,850 °C/min by adding 1 mL of 0.3 M sucrose solution maintained at 23, 37, or 50 °C. Warming the embryos at a rate of 27,300 or 36,700 °C/min resulted in a significantly lower level of development to the blastocyst stage (30.0 and 31.4%, respectively) than those warmed at a rate of 68,850 °C/min (58.8%, Fig. 1, Table S1). This level of development did not differ significantly from the success rate observed for fresh embryos (57.0%).

### Effect of warming rate and sample volume on survival of 1-cell rat embryos after cryopreservation

**Figure 2:**
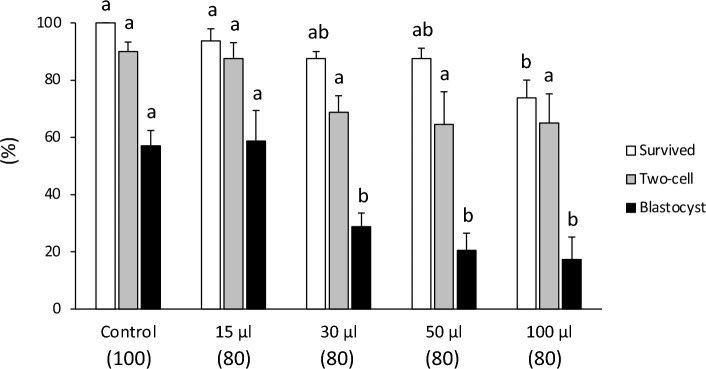
Survival and in vitro development of cryopreserved one-cell embryos in a sample volume of 15, 30, 50, or 100 μL, which were cooled using direct immersion in liquid nitrogen (LN_2_) at a rate of 7950, 7160, 6800 and 5830 °C/min, respectively, and warmed with sucrose solution at 50 °C. The warming rates were 68,850, 49,400, 44,500 and 35,480 °C/min, respectively. Each bar indicates the percentage of embryos surviving (open bars), developing to the two-cell stage (shaded bars), and developing to the blastocyst (closed bars) after cryopreservation (N = 8–10 experimental runs); numbers in parentheses indicate the number of one-cell embryos examined. Data are shown as means ± SEM. Different lowercase letters indicate a significant difference at *P* < 0.05.

When the sample volume of rat 1-cell embryos was increased from 15 to 100 μL, and the warming rate was decreased from 68,850 °C/min to 35,475 °C/min, the developmental ability of the embryos decreased from 58.8 to 17.3% (Fig. 2, Table S2). A sample volume of 15 μL resulted in rapid warming and high developmental potential; thus, the optimal sample volume for cryopreservation of 1-cell embryos was determined to be less than 15 μL.

### Survival of 2-, 4-, and 8-cell embryos, morulae, and blastocysts after small volume vitrification and rapid warming

**Figure 3:**
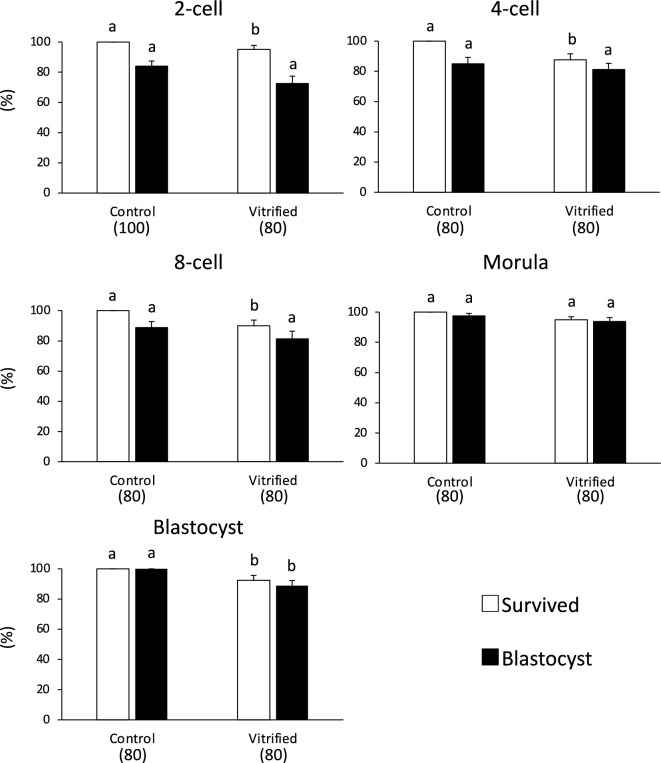
Survival and in vitro development of vitrified embryos at the 2-, 4-, and 8-cell embryos, morulae, and blastocysts in a sample volume of 15 μL, which were cooled using direct immersion in LN_2_ at a rate of 7950 °C/min, and warmed with sucrose solution at 50 °C at a rate of 68,850 °C/min. Each bar indicates the percentage of embryos surviving (open bars) and developing to the blastocyst stage (closed bars) after cryopreservation (N = 8–10 experimental runs); numbers in parentheses indicate the number of embryos examined at each developmental stage. Data are shown as means ± SEM. Different lowercase letters indicate a significant difference at P < 0.05. The developmental ability of blastocysts was assessed by determining whether they could develop into expanded blastocysts.

Rat embryos at other stages were cooled and warmed using the method developed for 1-cell embryos, including a 15 μL sample volume. The in vitro developmental success rate of cryopreserved embryos at these developmental stages ranged from 72.5 to 93.8%. No significant difference was observed compared with that of fresh, untreated embryos (84.0–100%, Fig. 3, Table S3).

### In vivo development of cryopreserved 1-cell rat embryos to term

**Figure 4:**
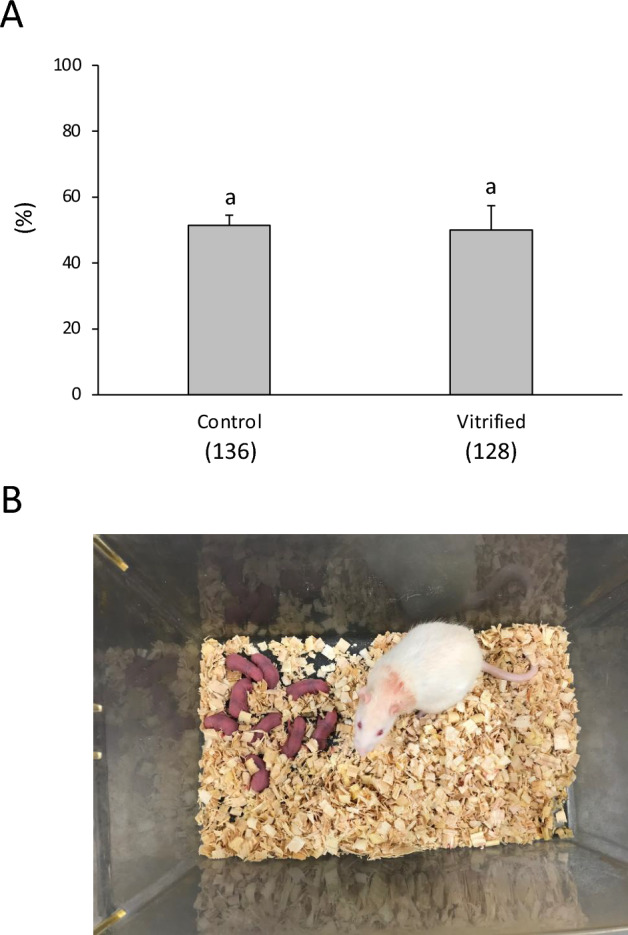
In vivo development of cryopreserved one-cell rat embryos. The one-cell embryos were vitrified with 5% PG for 10 min and PEPeS for 40 s. The embryos were warmed using 1 mL of sucrose solution at 50 °C. The cryopreserved or fresh embryos were transferred into the oviducts of pseudopregnant female rats. (**A**) Offspring survival. Numbers in parentheses indicate the number of one-cell embryos examined. Data are shown as means ± SEM. Different lowercase letters indicate a significant difference at *P* < 0.05. (**B**) Live pups derived from vitrified one-cell embryos.

The proportion of cryopreserved 1-cell rat embryos that developed to term following 1-cell embryo transfer was 50.0%, which did not differ significantly from that of the controls (51.4%, Fig. 4, Table S4).

## Discussion

In this study, we developed a method to cryopreserve rat embryos at all developmental stages using cryotubes and a small-volume vitrification procedure with rapid warming. High levels of subsequent rat embryonic development were confirmed at all developmental stages. This method can be applied in rat genome-editing research and strain preservation in gene banks.

Embryo cryopreservation is essential in many fields, including ART in human infertility treatment, cryobanks for laboratory animals, and livestock breeding. Several small devices or tools are currently used for the cryopreservation of mammalian embryos, including Cryotops, plastic straws, thin films, and minute loops. Cryotops are often used in ART to cryopreserve human oocytes and embryos; however, they are expensive when used for embryo cryopreservation of laboratory animals and only a limited number of embryos can be vitrified using Cryotops or the other small tools. Plastic straws are widely used as cryobanking containers and they have some advantages in the cryopreservation of mouse embryos. For example, plastic straws can hold several embryos and temperature changes are rapidly transmitted through the thin walls to the embryo-suspended solution. However, in plastic straws, it is difficult to describe a sample, and the samples are quite fragile to handle. Although Seita et al.^23^ demonstrated a high survival rate following cryopreservation of 1-cell rat embryos in Cryotops, cryotubes have several advantages over Cryotops in that they are easier to handle, more cost-effective, and can hold and preserve 100 embryos. It is possible to collect 40–60 embryos per female rat after superovulation with LH-RH, PMSG, and hCG, which is too many to cryopreserve in one Cryotop but not in one cryotube. Cryotubes are widely used in gene banks because they allow the processing of several embryos in a single sample and the samples can be easily handled. The vitrification of 2-cell-stage rat embryos using cryotubes yields high survival and developmental rates^24,25^, but this is not the case for 1-cell-stage embryos^26^. Since cryopreservation of embryos requires dehydration and permeation of a cryoprotectant, smaller cells are easier to cryopreserve. After fertilization, embryonic cells become smaller in size each time they cleave, making them easier to cryopreserve. Moreover, permeability of mammalian (mouse, bovine, and pig) morulae and blastocysts is quite high because of the expression of aqua-glyceroporins in the plasma membrane^27–29^. As a result, the cryopreservation protocol for 1-cell embryos developed in this study can be applied to rat embryos at other developmental stages.

**Figure 5:**
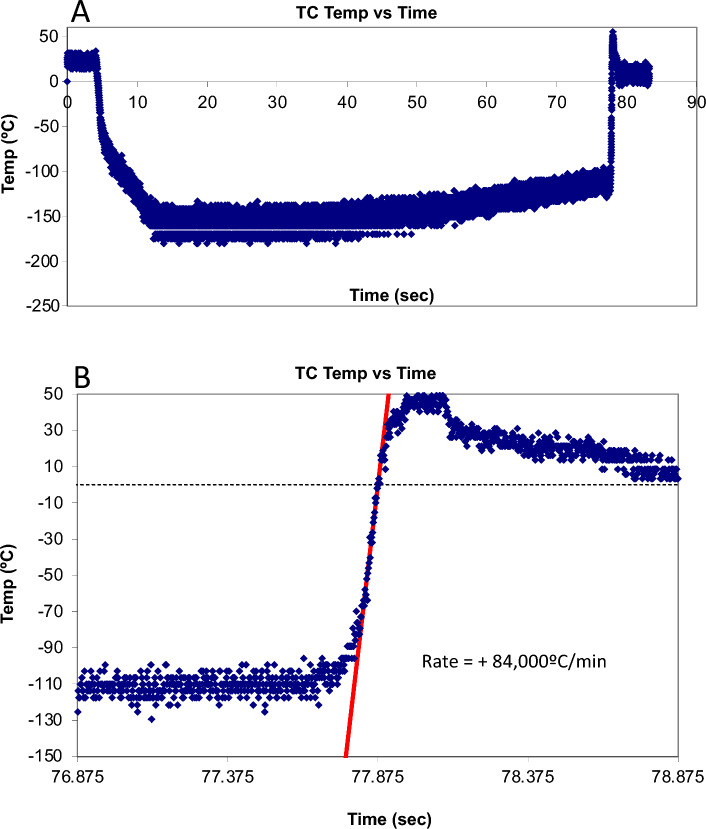
(**A**) Thermal cycle oscilloscope trace of a 15 μL aqueous sample (PEPeS) in vitrification solution^24^ in a cryotube equipped at the bottom with a 50 μm Cu-constantan thermocouple. The cryotube sample was rapidly moved from room-temperature air (~ 23 °C) to LN_2_ and held for 5 s, and then held in air at 23 °C for 1 min before warming by the addition of 1 mL of sucrose solution at 50 °C. (**B**) Details of events during warming when the sample was warmed by adding 1 mL of sucrose solution at 50 °C. A straight line visually fit to the data revealed a warming rate of 84,000 °C/min. The temperature sampling rate was 500 readings/sec.

Since mouse embryos are sensitive to heat stress^30^, the development of blastocysts from 2-cell mouse embryos was disturbed by treatment at 41 °C for 2 h, but not by treatment at 40 °C for 1 h^31^. Therefore, it is possible that rat embryos are also damaged by treatment at a high temperature. In this study, we vitrified rat embryos and warmed them using a sucrose solution at 50 °C. Although the sample reached 50 °C (Fig. 5), it was less than 0.3 s, and the temperature decreased to less than 37 °C, which did not result in any damage to the embryos. In fact, when rat 1-cell embryos were exposed to Rat-KSOM at 50 °C for 10 s, their developmental rate (63.3 ± 9.3%, N = 5, 28 of 50 embryos) to blastocysts in vitro was similar to that of fresh embryos (57.0 ± 5.4%, N = 10, 57 of 100 embryos). Therefore, rat embryos were not sensitive to the short exposure at a high temperature.

A question in this study was whether the cryopreservation solution was vitrified or frozen. We confirmed by naked-eye observation that a small part of the solution (a mixture of 5 μL of 5% propylene glycol solution and 10 μL of PEPeS) turned white when cooled in LN_2_. Furthermore, we conducted differential scanning calorimetry (DSC, Perkin Elmer DSC8500) analysis of the abovementioned mixture. When the mixture (15 μL) was cooled to − 100 °C (the lowest limit of the DSC system) at a rate of 200 °C/min (highest cooling rate of the DSC system), exotherms due to freezing of the medium were detected around − 60 °C (data not shown). However, at the very least, the rat embryos survived cryopreservation and thawing, and they were vitrified without the formation of ice crystals.

During warming, after discarding liquid nitrogen from the cryotube, the cryotube was kept at 23 °C (room temperature) to pass through the temperature range in which fractures occur (− 135 °C to − 110 °C) due to slow warming. When the sample volume was 15–30 μL, the temperature reached − 104.4 °C and − 106.5 °C by holding the cryotube for 1 min at 23 °C (room temperature) (Figure S1). However, when the sample volume increased to 50–100 μL, the temperatures were − 113.8 °C and − 119.8 °C. As the sample volume increased from 15 μL to 30 μL, no significant decrease was observed in survival and a significant decrease was observed in the potential to develop blastocysts (Fig. 2), which was thought to be due to damage by recrystallization. However, a significant decrease in survival was observed when the sample volume reached 100 μL, which may be because of fracture damage.

The cryotube cryopreservation method used in this study exhibits high developmental potential, even for 1-cell embryos. Most technicians could perform this method in a short period of time without the use of expensive or fine tools, and without the need for embryo manipulation. In fact, upon verification by ARK Resources Co., Ltd., similar results were obtained by technicians with different levels of embryo manipulation proficiency. Our protocol can also be applied to other strains of rats. For example, we confirmed that the Long-Evans strain of rat can develop into blastocysts in vitro after vitrification and warming using the described protocol, with rates similar to those of fresh controls (data not shown).

In conclusion, we developed an efficient cryotube method for the cryopreservation of rat embryos at all developmental stages by small-volume vitrification procedure and rapid warming in cryotubes. Embryos were suspended in a minimal amount of cryopreservation solution containing a relatively low concentration of permeating cryoprotectant, rapidly immersed in LN_2_, moderately warmed to approximately − 110 °C, and then rapidly warmed by adding a sucrose solution warmed to 50 °C. This cryopreservation method has several potential applications in biological and biomedical research, including genome editing research, while contributing to the understanding of the mechanisms of human disease and physiology.

## Materials and methods

### Animals

Male and female Iar: Wistar-Imamichi rats (10–16 weeks old; Japan SLC Inc., Shizuoka, Japan) were housed in an environmentally controlled room (24 ± 3 °C and 50 ± 10% humidity), and subjected to a 12 h light (07:00–19:00)/12 h dark cycle. All animal experiments were approved by the Animal Experimentation Committee at Akita University (ID: a-1-0337) and performed in accordance with ARRIVE guidelines (Animal Research: Reporting of In Vivo Experiments) and with the guidelines of the committee at Akita University. This study was approved by the ethics committee of Akita University.

### Collection of embryos and embryo transfer

Rat embryos at the 1-, 2-, and 8-cell stage, as well as morulae and blastocysts, were used in this study. Female rats subjected to estrus cycle synchronization were injected intraperitoneally with [des-Gly10, D-Ala6]-LH-RH ethylamide acetate salt hydrate (LH-RH; 0.04 mg dissolved in 200 μL of saline; Sigma-Aldrich, St. Louis, MO, USA)^32^. After 48 h, female rats (10–16 weeks old) were intraperitoneally injected with pregnant mare serum gonadotropin (PMSG, 150 IU/kg, ASKA Animal Health Co. Ltd, Tokyo, Japan) and human chorionic gonadotropin (hCG, 75 IU/kg, ASKA Animal Health Co. Ltd, Tokyo, Japan), administered 48 h apart. The same strain of mature male rats (12–20 weeks old) after hCG injection.

To collect 1- and 2-cell embryos, oviducts of mated animals were flushed with M2 medium (Millipore Corporate Headquarters, Billerica, MA, USA) 25 and 44 h after injection with hCG, respectively. One-cell embryos were freed from cumulus cells by suspending them in M2 medium containing hyaluronidase (0.5 mg/mL), followed by washing with fresh M2 medium. To obtain 4- and 8-cell embryos, morulae and blastocysts, 2-cell embryos were cultured to the respective developmental stage in vitro in Rat KSOM (ARK Resource Co. Ltd, Kumamoto, Japan) for 24, 48, 72, and 96 h, respectively. This was necessary because it is difficult to collect these stages by flushing the oviducts and uterine horn at 78 and 92 h after hCG injection, respectively. Collected embryos were suspended in Rat KSOM (200 μL)^33^ at 37 °C in 5% CO_2_ in air.

### Composition of cryopreservation solution and warming solution

Embryos were cryopreserved using PEPeS (10% v/v propylene glycol, 30% v/v ethylene glycol, 0.3 M sucrose, and 20% v/v Percoll [GE Healthcare, Uppsala, Sweden] in phosphate-buffered saline 1 [PB1]) developed by Eto et al.^24^ for the cryopreservation of rat 2-cell embryos^34,35^. To warm cryopreserved embryos, PB1 solution containing 0.3 M sucrose (hereafter, 0.3 M sucrose solution) was used. PEPeS was purchased from ARK Resource Co., Ltd., and the other reagents used to prepare the solution were purchased from FUJIFILM Wako Pure Chemical Corporation (Osaka, Japan). The compositions of the cryopreservation solutions used in this study are listed in Table 1.

**Table 1:** Composition of cryopreservation solutions.

Solution	Propylene glycol (PG; %, v/v)	Ethylene glycol (%, v/v)	Percoll	Sucrose (M)	Osmolality (mol/kg water)
5% PG	5.0	0.0	0.0	0.0	0.99
PEPeS	10.0	30.0	20.0	0.3	9.99
5% PG (5 μL) + PEPeS (10 μL)	8.2	20.0	13.3	0.2	5.97

### Cryopreservation procedure

Embryos were suspended in PB1 (10–50 μL), which included 5% propylene glycol, at room temperature (23 °C) with three sequential washes for 10 min. A total of 10–20 embryos in a 5-μL drop of medium was then placed to the bottom of a cryotube (MS-4501W; Sumitomo Bakelite Co., Ltd., Tokyo, Japan) and cooled at 0 °C for 1 min. Thereafter, vitrification solution (10 μL), precooled to 0 °C, was added to the cryotube, and incubation continued for 40 s before cooling by direct immersion of the cryotube in liquid nitrogen (LN_2_). Thus, 11 min and 40 s elapsed from the first exposure of the embryos to the cryopreservation solution until cooling in LN_2_ was initiated. The effect of the warming rate on subsequent development was tested in one experimental group. Five, 10, 16.7, or 33.3 μL of PB1 with 5% propylene glycol included 10–20 embryos were transferred to cryotubes with a laboratory pipette (Gilson Inc., Middleton, WI, USA), and 10, 20, 33.3, or 66.7 μL of vitrification solution was added, respectively. As a result, cryopreservation and warming were carried out using total sample volumes of 15, 30, 50, and 100 μL.

### Testing of various cooling rates and warming rates of the cryopreservation solution

Determination of cooling and warming rates was performed as described by Kleinhans et al.^36^. More specifically, the junction of a 50-μm Type T (copper-Constantan) thermocouple was fixed to the bottom of a cryotube with glue (Aron Alpha, Osaka, Japan), and the distal wires were attached to a computer-oscilloscope system. A droplet of PEPeS solution (15, 30, 50, or 100 μL) was placed over the junction and a wide range of cooling and warming rates were investigated (Table 2). The samples were cooled via direct immersion in LN_2_. Warming rates were investigated by subjecting cryotube samples to one of three warming temperatures using the following procedure. First, the cap of a cryotube was removed and the LN_2_ was discarded. Second, each sample was gently warmed by holding it in air at 23 °C for 1 min to prevent fracture damage, which can occur at approximately − 135 °C to − 110 °C^37–39^. Finally, each sample was warmed by adding 1 mL of a 0.3 M sucrose solution, previously warmed to 23, 37, or 50 °C, and the resulting warming rate (°C/min) was determined using the thermocouple (Table 2). An oscilloscope trace of one cooling and warming run is shown in Fig. 5A. The cooling rates ranged from 5825 to 7950 °C/min based on the time taken to cool the solution from 0 to about − 50 °C (Table 2), because IIF during cooling occurs around − 35 °C^40^. Warming rates were calculated based on the time taken for the temperature to increase from − 100 °C to approximately 0 °C (Fig. 5B). The range included − 80 °C to − 35 °C, the temperature range where recrystallization occurs in mouse oocytes, and because recrystallization of ice ceases above approximately − 35 °C^3^. The thermocouple was omitted from the runs when embryo survival was being measured after the various treatments. Moreover, temperature change at the bottom of the cryotube containing 15 μL of the vitrification solution (PEPeS) during warming was determined by monitoring each second using a digital thermometer (Figure S1, CENTER SE-309, Satoshoji Co., Kawasaki, Japan).

**Table 2:** Cooling and warming conditions.

Volume of 5% PG^a^ + PEPeS (μL)	Cooled by	Cooling rate ± SEM (°C/min)	Temperature of sucrose (0.3 M) warming solution (1 mL) (°C)	Warming rate ± SEM (°C/min)
15	LN_2_	7950 ± 210 (*N* = 8^b^)	23	29,930 ± 2180 (*N* = 8)
			37	38,780 ± 2010 (*N* = 8)
			50	68,850 ± 5670 (*N* = 8)
30		7150 ± 105 (*N* = 8)	50	49,400 ± 2860 (*N* = 8)
50		6800 ± 320 (*N* = 8)	50	44,500 ± 3520 (*N* = 8)
100		5830 ± 250 (*N* = 8)	50	35,480 ± 1320 (*N* = 8)

^a^PG, propylene glycol; PEPeS, 10% v/v PG, 30% v/v ethylene glycol, 0.3 M sucrose, and 20% v/v Percoll in phosphate-buffered saline); LN_2_, liquid nitrogen.

^b^Refers to the number (*N*) of experimental run.

### Assessment of the developmental ability of cryopreserved rat embryos

**Figure 6:**
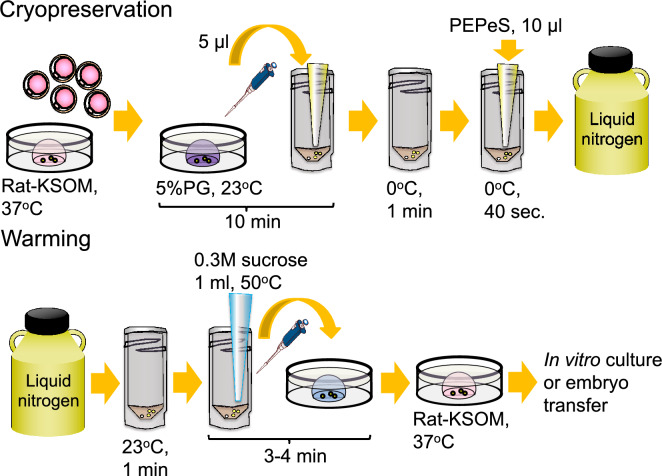
Cryopreservation and warming procedure for rat embryos developed in this study.

After warming, the 0.3M sucrose solution containing the rat embryos was transferred to a plastic dish at 23 °C (room temperature), kept for 3–4 min, and then transferred to pre-equilibrated Rat KSOM at 37 °C in 5% CO_2_. Figure 6 shows the cryopreservation and warming processes. The morphologically normal embryos were cultured in Rat KSOM at 37 °C in 5% CO_2_ in air and the medium was changed daily. The in vitro survival rate of the embryos was assessed based on the cleavage and development rates of the blastocysts. Blastocysts were assessed by determining whether they could develop into expanded blastocysts. In one experimental group, embryo transfer was used to assess the ability to develop to term. The cryopreserved 1-cell embryos were incubated in Rat KSOM for 1 h before embryo transfer. Eight cryopreserved or fresh, untreated embryos were transferred into the oviducts of pseudopregnant female rats of the same strain. To induce pseudopregnancy, female rats that had been injected with LH-RH 4 d previously were introduced to vasectomized male rats on the day before embryo transfer. The female rats with transferred embryos were subjected to caesarean section on day 19 or 20 to determine the number of normal fetuses. In one experimental group, the offspring were confirmed to be derived from natural birth. Surgical operations such as vasectomies and embryo transfer were performed under 2–3% isoflurane (Pfizer Inc., New York, NY, USA) inhalation anethesia.

### Statistical analysis

Data are reported as the mean number of embryos (%) ± standard error of the mean (SEM). The n values represent the number of experimental runs. Significant differences were determined using one-way ANOVA and the Tukey–Kramer Multiple comparison test. Statistical significance was set at *P* < 0.05. The survival of fresh, untreated embryos was 100% and the SEM was 0 or the test was conduct for two experimental groups; thus, one-way ANOVA was not appropriate. In those cases, significant differences were determined using Fisher’s exact test.

### Supplementary Information

Supplementary Information.

## Data Availability

All data generated or analyzed during this study are included in this published article and its supplementary file.
