# Supplementary material for: Cryopreservation of rat embryos at all developmental stages by small-volume vitrification procedure and rapid warming in cryotubes
Source: Sci Rep. 2023 Nov 27;13:20903. doi: 10.1038/s41598-023-47394-0 (PMC10684866; doi:10.1038/s41598-023-47394-0)
Supplement: Supplementary file 1 — Supplementary Information. [file 41598_2023_47394_MOESM1_ESM.pdf]

Supplementary Information

Title

Cryopreservation of rat embryos at all developmental stages by small-volume vitrification procedure and rapid warming in cryotubes

Authors

Shinsuke Seki[1*], Toshiaki Kawabe[2], Wataru Yamazaki[1], Kazuaki Matsumura[3], Takanori Oikawa[1], Takahiro Obata[1], Misako Higashiya[1], Megumi Yano[1], Tomoo Eto[4*]

[1] Experimental Animal Division, Bioscience Education and Research Support Center, Akita University, 1-1-1 Hondo, Akita, Akita 010-8543, Japan
[2] ARK Resource Co., Ltd., 456 Osozu, Misato-machi, Shimomashiki-gun, Kumamoto 861-4401, Japan
[3] School of Materials Science, Japan Advanced Institute of Science and Technology, 1-1 Asahi-dai, Nomi, Ishikawa 923-1292, Japan
[4] Central Institute for Experimental Animals, 3-25-12 Tonomachi, Kawasaki-ku, Kawasaki 210-0821, Japan

Correspondence should be addressed to S.S. (email: sseki@gipc.akita-u.ac.jp) and T.E. (email: etoh@ciea.or.jp)

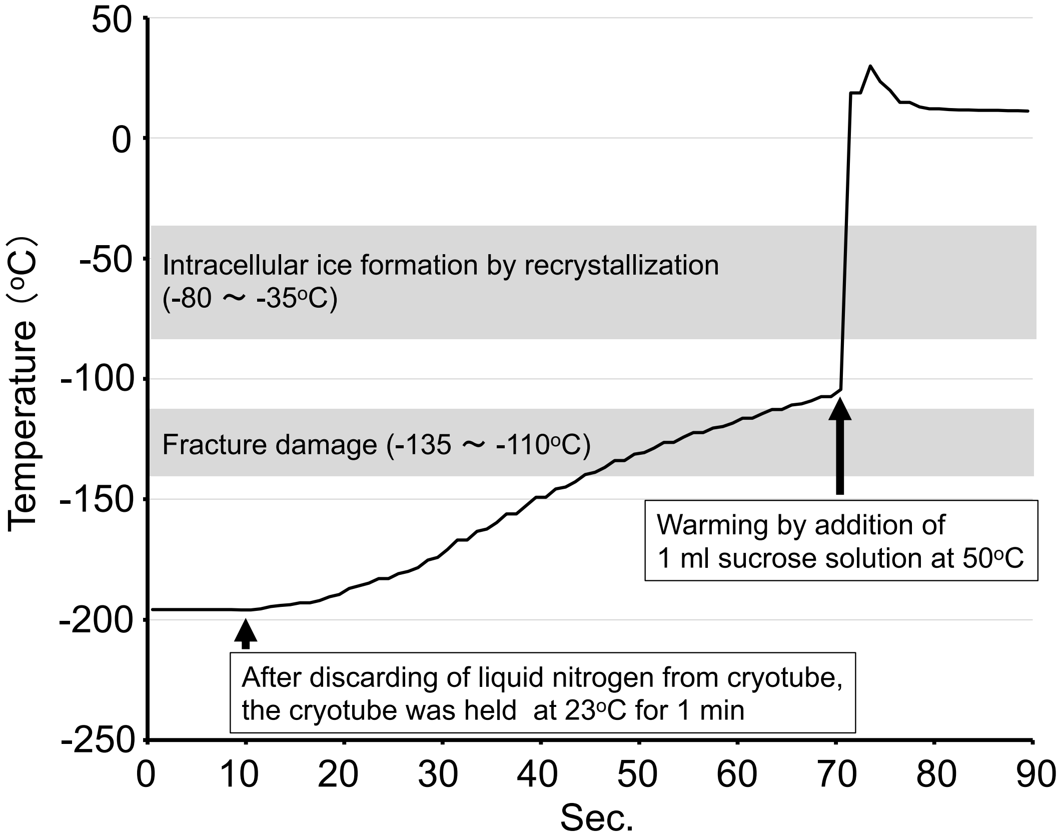

Figure S1 Temperature change at the bottom of cryotube includes 15 μl vitrification solution (PEPeS) during warming. Liquid nitrogen was discarded from cryotube (at 10 sec.), the cryotube was kept at 23°C room temperature for 1 min to pass through the temperature range (from -135 to -110°C) which fracture damage occurs by slow warming, and the vitrification solution was warmed rapidly by adding 1ml sucrose solution at 50°C into the cryotube (at 70 sec.) to avoid intracellular ice formation by recrystallization. The temperature was monitored each second using a digital thermometer (CENTER SE-309, Satoshoji Co., Kawasaki, Japan).

Table S1 Survival and *in vitro* development of vitrified one-cell embryos (15 μL sample volume) warmed with sucrose solution at 23, 37, or 50°C.

Cryopreservation	Sample volume (μL)	Cooling rate (^{o}C/min)	Temperature of sucrose warming solution	Warming rate (^{o}C/min)	No.of embryos			
					Cryopreserved or cultured as control (%)	Survived (%)	Developed to the 2-cell stage (%)	Developed to the blastcyst stage (%, N)
+	15	7,950	23°C	29,930	80	77 (96.7 ± 2.0%)	66 (80.0 ± 3.5%)	23 (30.0 ± 6.1%, N = 8)
+	15	7,950	37°C	38,780	70	64 (91.4 ± 3.4%)	56 (80.0 ± 3.8%)	22 (31.4 ± 5.1%, N = 7)
+	15	7,950	50°C	68,850	80	75 (93.8 ± 4.2%)	70 (87.5 ± 5.6%)	47 (58.8 ± 10.6%, N = 8)
- (non-cryopreserved)	N/A	N/A	N/A	N/A	100	100 (100 ± 0%)	90 (90.0 ± 3.3%)	57 (57.0 ± 5.4%, N = 10)

Table S2 Survival and *in vitro* development of cryopreserved one-cell embryos in a sample volume of 15, 30, 50, or 100 μL.

Cryopreservation	Sample volume (μL)	Cooling rate (°C/min)	Temperature of sucrose warming solution	Warming rate (°C/min)	No.of embryos			
					Cryopreserved or cultured as control (%)	Survived (%)	Developed to the 2-cell stage (%)	Developed to the blastcyst stage (%, N)
+	15	7,950	50°C	68,850	80	75 (93.8 ± 4.2%)	70 (87.5 ± 5.6%)	47 (58.8 ± 10.6%, N = 8)
+	30	7,150	50°C	49,400	80	70 (87.5 ± 2.5%)	55 (68.8 ± 5.8%)	23 (28.8 ± 4.8%, N = 8)
+	50	6,800	50°C	44,500	80	70 (87.5 ± 3.7%)	48 (64.5 ± 11.4%)	15 (20.6 ± 6.0%, N = 8)
+	100	5,830	50°C	35,480	80	59 (73.8 ± 6.3%)	44 (65.0 ± 10.2%)	10 (17.3 ± 7.9%, N = 8)
- (non-cryopreserved)	N/A	N/A	N/A	N/A	100	100 (100 ± 0%)	90 (90.0 ± 3.3%)	57 (57.0 ± 5.4%, N = 10)

Table S3 Survival and *in vitro* development of vitrified 2-, 4-, and 8-cell embryos, morulae and blastocysts in a sample volume of 15 μL.

Developmental stage	Cryopreservation	No.of embryos		
		Cryopreserved or cultured as control (%)	Survived (%)	Developed to the blastcyst stage (%, N)
2-cell	+	80	76 (95.0 ± 2.7%)	58 (72.5 ± 4.9%, N = 8)
	- (non-cryopreserved)	100	100 (100 ± 0%)	84 (84.0 ± 3.4%, N = 10)
4-cell	+	80	70 (87.5 ± 4.1%)	65 (81.3 ± 4.0%, N = 8)
	- (non-cryopreserved)	80	80 (100 ± 0%)	68 (85.0 ± 4.2%, N = 8)
8-cell	+	80	72 (90.0 ± 2.0%)	65 (81.3 ± 2.0%, N = 8)
	- (non-cryopreserved)	80	80 (100 ± 0%)	71 (88.8 ± 4.0%, N = 8)
Morula	+	80	76 (95.0 ± 1.9%)	75 (93.8 ± 2.6%, N = 8)
	- (non-cryopreserved)	80	80 (100 ± 0%)	78 (97.5 ± 1.6%, N = 8)
Blastocyst	+	80	74 (92.5 ± 3.1%)	71 (88.8 ± 3.5%, N = 8)
	- (non-cryopreserved)	80	80 (100 ± 0%)	80 (100 ± 0%, N = 8)

Table S4 *In vivo* development of cryopreserved one-cell rat embryos.

No.of embryos			No. of recipients	No. of embryos	
Cryopreservation	Cryopreserved or cultured as control (%)	Survived (%)		Transferred	Developed to offsprings (%)
+	160	144 (90.0 ± 2.7%)	8	136	64 (50.0 ± 7.4%, N = 8)
- (non-cryopreserved)	136	136 (100 ± 0%)	8	144	70 (51.4 ± 3.1%, N = 8)
